# Adsorptive Removal of Rhodamine B Dye Using Carbon Graphite/CNT Composites as Adsorbents: Kinetics, Isotherms and Thermodynamic Study

**DOI:** 10.3390/ma16031015

**Published:** 2023-01-22

**Authors:** Sabrine Zghal, Ilyes Jedidi, Marc Cretin, Sophie Cerneaux, Makki Abdelmouleh

**Affiliations:** 1 Laboratory of Materials Science and Environment (LMSE), Faculty of Science of Sfax, University of Sfax, Rte. Soukra Km 4, Sfax 3000, Tunisia; 2 Department of Engineering, College of Engineering and Technology, University of Technology and Applied Science, Al Jamiaa Street, Suhar 311, Oman; 3 Institut Européen des Membranes, IEM—UMR 5635, ENSCM, CNRS, Université de Montpellier, 34095 Montpellier, France

**Keywords:** carbon nanotubes, Rhodamine B dye, adsorption, kinetic, isotherm, thermodynamic

## Abstract

The study of the adsorption efficiency of new carbon/CNT composites was undertaken to remove a cationic dye, Rhodamine B (RhB), from dye-contaminated wastewater. Indeed, we investigated the effect of different experimental parameters such as time, initial concentration of dye and temperature on the adsorption of RhB by the carbon composites (KS44-0 and KS44-20). The results showed that the adsorption uptake increased with the initial concentration and solution temperature while maintaining a relatively constant pH. The presence of the carbon nanotubes provided more active sites for dye removal and improved the adsorption behavior of Rhodamine B dye. The analysis of the experimental data was conducted using model equations, such as Langmuir, Freundlich and Temkin isotherms. As regards the Freundlich isotherm model, it was the best fit for the equilibrium data obtained from the experiments. The applicability of the pseudo-second-order equation could be explained assuming that the overall adsorption rate is limited by the rate of adsorbate transport that occurs on the pore surfaces of adsorbents. Furthermore, the intraparticle diffusion and Bangham models were used to investigate the diffusion mechanism of RhB absorption onto carbon composites. They showed that multiple adsorption stages occurred simultaneously via pore surface diffusion. Concerning the thermodynamic parameters (∆G°, ∆H°, and ∆S°), they were calculated and explained in the mean of the chemical structure of the adsorbate. Negative standard Gibbs free energy change values (ΔG°_ads_) at all temperatures suggested that the adsorption process was spontaneous, and the positive values of the standard enthalpy change of adsorption (∆H°_ads_) revealed the reaction to be endothermic. The values of standard enthalpy (ΔH°_ads_) and activation energy (E_a_) indicated that the adsorption process corresponds to physical sorption. The mechanisms for the removal of Rhodamine B dye from wastewater using carbon composite were predicted. RhB is a planar molecule that is readily adsorbed, in which adsorbed molecules are bound by hydrophobic or other weak interactions due to the π-π interactions between the dyes’ aromatic backbones and the hexagonal skeleton of graphite and carbon nanotubes. Thus, the graphite carbon/carbon nanotube composite is believed to play a major role in organic pollutant reduction.

## 1. Introduction

Synthetic dyes represent a relatively large group of organic chemical compounds, virtually found in all areas of our daily life. They make up the largest family of dyestuffs, accounting for more than half of global production. They are widely used, particularly in the dyeing factories of textile fibers, leather articles, stationary products, plastics, etc. [1]. Wastewater from the textile sector represents a real challenge in terms of dye removal since it frequently contains diverse colors and heavy metals. Thanks to their unique physico-chemical characteristics, reactive dyes are the favored choice in the textile industry [2]. Most dye molecules are highly colored organic compounds due to the presence of chromophore and its fixed property to the acid or basic auxochromic groups (such as OH, SO_3_H, NH_2_, NR_2_, etc.). These dyed molecules, which are widely used to impact color various substances, can be dissolved in an adequate organic solvent or incorporated into a solid matrix by physical and chemical interactions (for instance, the incorporation of groups that reduce solubility (e.g., amide groups), or the lake formation of insoluble salts of carboxylic or sulfonic acids groups) [3]. Given that these dyes have chemical structures that contain amino and carboxyl groups and are therefore highly poisonous, mutagenic, and carcinogenic, excessive quantities of these dyes in wastewater constitute a serious hazard to human health and the aquatic ecology [4]. The hazard presented by the increasing amount of dye used on a daily basis, especially on our ecosystem, has prompted a serious quest for more efficient, low-cost adsorbents.

According to earlier studies, Rhodamine B may be removed from aqueous solutions using activated carbon made from almond shells [5], and that from Palm shell was successfully tested as an adsorbent [6]. Furthermore, raw Irvingia gabonenses (dika nut) (DN) and its acid-treated form (ADN) increased the absorption of Rhodamine B (RhB) dye from aqueous solutions [7]. Activated carbon obtained from the pericarp of rubber fruit was more efficient in optimizing the adsorption process in comparison with commercial activated carbon [8]. The effects of several experimental factors such as contact time, dye concentration, activated carbon quantity, and temperature were investigated. The equilibrium results suggested that the Langmuir model was better suited to represent Congo red adsorption and had high reusability potential.

Despite the fact that there are several challenges with graphene-based composite and carbon nanotube synthesis, such as separation/dispersion, controlled manipulation, dispersion and defect-free manufacturing, graphene and carbon nanotubes are one of the most extensively utilized adsorbents. This is due to their considerable adsorption capacity towards the removal of toxic inorganic and organic contaminants from water and wastewater to reduce their environmental impact [9,10].

Our most recent publication, which uses the ferrocene chemical, describes a one-step approach for creating porous carbon graphite/carbon nanotube composites. The latter acted as both a carbon source and catalyst precursor agent for the in situ growth of carbon nanotubes (CNTs), and both phenolic resin and graphite powder were used as carbon precursors for the carbonaceous material synthesis [11]. We found that carbon nanotubes grown inside a porous carbon structure significantly improved the material’s adsorption performance toward the synthetic Acid Orange 7 azo-dye (AO7) when tested as an adsorbent for the treatment of wastewater containing persistent organic pollutants. This was in contrast to the same material prepared without CNTs. [12].

In the present research work, we mainly concentrated on the application of these graphite carbon/carbon nanotube composites for the adsorption-based removal of organic dye from aqueous systems. To investigate the feasibility of our approach, we selected Rhodamine B (RhB) as a model pollutant for a long-lasting dye molecule to explore the potential use of our porous carbon composites as cost-effective carbon adsorbents through the adsorption method. Therefore, this strategy is expected to enhance the adsorption capacity of the resulting porous carbon by avoiding carbon nanotubes’ growth within the porosity of the composites materials while maintaining the efficiency of RhB dye pollutants’ adsorption. Overall, this study has made progress in proving the viability of synthesized carbon composites in environmental remediation and wastewater organic dye treatment. The effect of various operational parameters (contact time, initial dye concentration, temperature and adsorbent nature) on the dye removal efficiency onto carbon composites was examined. The experimental data were also interpreted using a variety of adsorption kinetics, processes, isotherm models and thermodynamics studies.

## 2. Materials and Methods

### 2.1. Materials

Two different carbon composites were elaborated based on our previous research [11]: one without ferrocene powder named KS44-0, and another containing a weight ratio of 20 wt.% of ferrocene which was added as a catalyst for the carbon nanotubes’ (CNTs’) growth, named KS44-20’.

Rhodamine B (Basic violet 10, Tetraethylrhodamine, [9-(o-Carboxyphenyl)-6-(diethylamino)-3H-xanthen-3-ylidene] diethylammonium chloride), (C_28_H_31_ClN_2_O_3_, 80%) was obtained from BIO BASIC. Benzenesulfonic acid sodium salt (4-(2-Hydroxy-1-naphthylazo)) (C_6_H_5_SO_3_Na), anhydrous sodium sulphate (Na_2_SO_4_, 99%), ferrous sulphate heptahydrate (FeSO_4_. 7H_2_O, >99%) and sulfuric acid (H_2_SO_4_, 98%) were purchased from Sigma-Aldrich.

### 2.2. Adsorption Experiments

The adsorption experiments were performed using Rhodamine B (RhB) as adsorbate and carbon composite materials as adsorbents. An amount of 0.6 g of carbon composites was dispersed through a soaking method into 30 mL RhB aqueous solutions of different initial concentrations which varied from 2.5 mg/L to 20 mg/L. All the experiments were carried out by adjusting the pH at acidic pH (pH = 3) using H_2_SO_4_ (1 M), over a temperature range of 20 °C (±2 °C) to 60 °C (±2 °C). The absorbance of RhB dye was measured using a spectrophotometer, Jenway 6300 (Barioworld Scientific Ltd., Dunmow, UK), at a wavelength of 555 nm, and the adsorption percentages of RhB were calculated as follows [13]:

(1) $${\mathrm{RhB}}_{\mathrm{ads}\;}\left(\%\right)=\frac{A_{i}{\;-\;A}_{e}}{A_{i}}\;\times \;100$$

where ${\mathrm{RhB}}_{\mathrm{ads}\;}\left(\%\right)\;$is the adsorption percentage uptake, Ai is the initial absorbance of RhB and Ae the equilibrium absorbance of RhB.

The amounts of RhB adsorbed onto the unit amount of carbon composites (mg.g^−1^) q_e_ were calculated according to Equation (2) [13]:

(2) $$q_{e}=\frac{\left(C_{0}-{\;C}_{e}\right)\;V}{W}$$

with C_0_ and C_e_ being the initial and equilibrium concentrations of RhB (mg.L^−1^), respectively. V represents the volume of the RhB solution (L), and W is the weight of the carbon composites (g).

To determine the concentration of Rhodamine B, a calibration curve was established at the maximal absorbance wavelength of 555 nm. A graphical representation of the calibration curve and the chemical structure of a Rhodamine B dye molecule are shown in Figure S1.

## 3. Results

### 3.1. Kinetics Study

#### 3.1.1. Effect of Initial Dye Concentration

**Figure 1:**
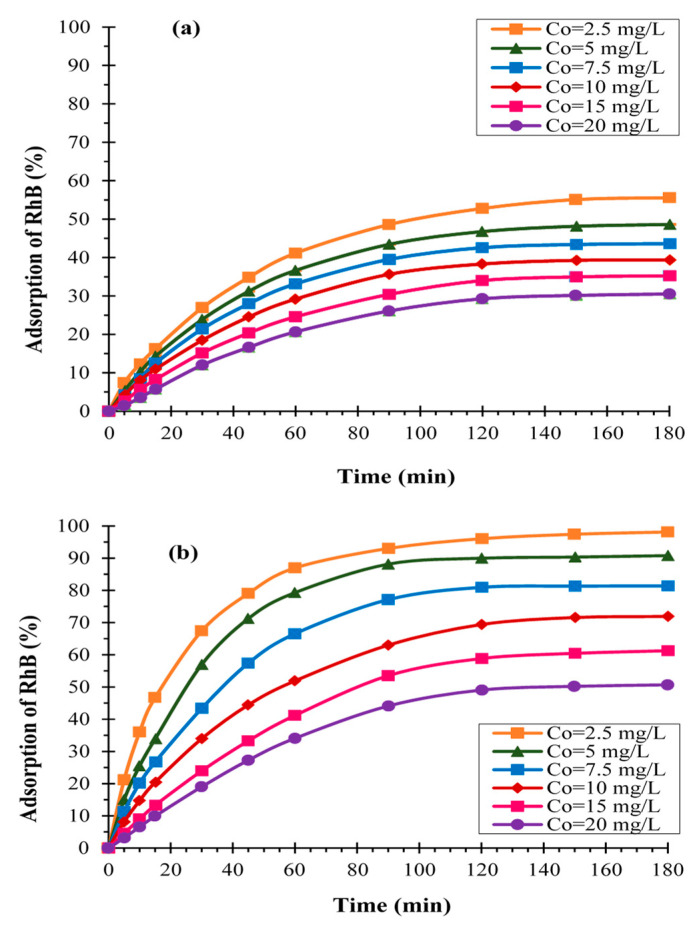
Evolution of the adsorption of RhB dye (%) at different initial concentrations on (**a**) KS44-0 and (**b**) KS44-20 (T = 20 °C, pH = 3 and time = 3 h).

**Figure 2:**
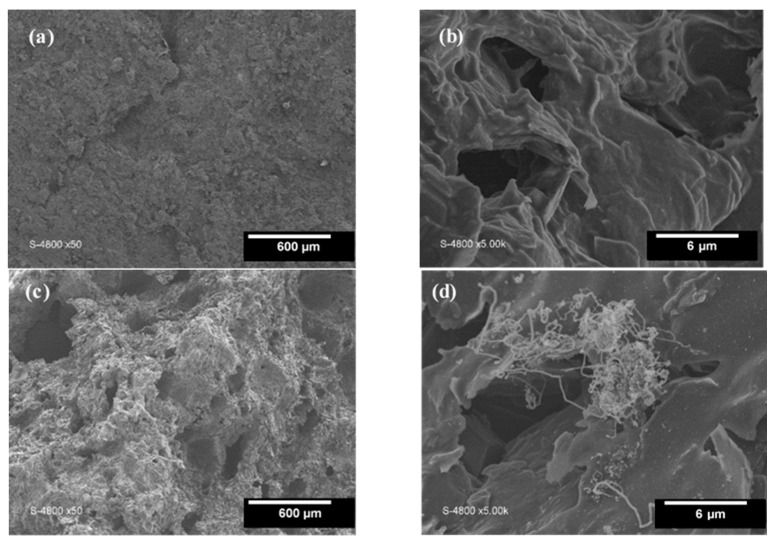
SEM micrographs at different scales of the material: (**a**,**b**) carbon graphite (KS44-0) and (**c**,**d**) carbon graphite/CNT composite (KS44-20).

Figure 1 depicts the adsorption kinetics of RhB onto carbon graphite (KS44-0) and carbon graphite/CNT (KS44-20) composite materials as a function of time (t), expressed as a percentage, for different RhB initial concentrations (C_0_) in the range of 2.5–20 mg/L. The results revealed that the adsorption capacity uptake of RhB increased onto carbon composites with the increase in the initial dye concentration. As shown in Figure 1, the dye adsorption process of RhB onto KS44-0 and KS44-20 reached an equilibrium after 120 min of contact time for all the initial concentrations tested. At the lower initial concentration of 2.5 mg/L, the dye adsorption efficiency values of RhB were 98% and 55% for KS44-20 and KS44-0, respectively. However, at a 20 mg/L initial RhB concentration, the adsorption quantity on the two supports was divided by 2. In fact, the increase in the initial RhB concentration favored the adsorbent–adsorbate interactions and led to the saturation of active sites accessible at the inner and outer surfaces of the porous carbon composites. This is because the driving force for mass transfer (i.e., the gradient of concentration) increased with the increase in the initial RhB dye concentration [14]. Actually, the existence of carbon nanotubes that expanded dramatically within the substance is the reason behind the higher RhB adsorption capacity and effectiveness seen with KS44-20 (Figure 2d). Carbon nanotubes with various structures that appeared all over the sample, on the inner faces of the pores and onto the surface, drastically changed the surface properties, thus leading to significant changes in the specific surface area and porosity of the KS44-20 composite compared to the KS44-0 composite (Figure 2b,d) [11,12].

Additionally, morphological and structural analyses of carbon composite materials, as well as information pertaining to their surfaces, showed that KS44-20 had a more significant porous structure than that that of KS44-0 (Figure 2a,c). The pore shape difference between KS44-0 and KS44-20 was well-illustrated by scanning electron microscopy, BET the surface areas and mercury porosimetry studies (Table 1) [11,12].

#### 3.1.2. Effect of Temperature

**Figure 3:**
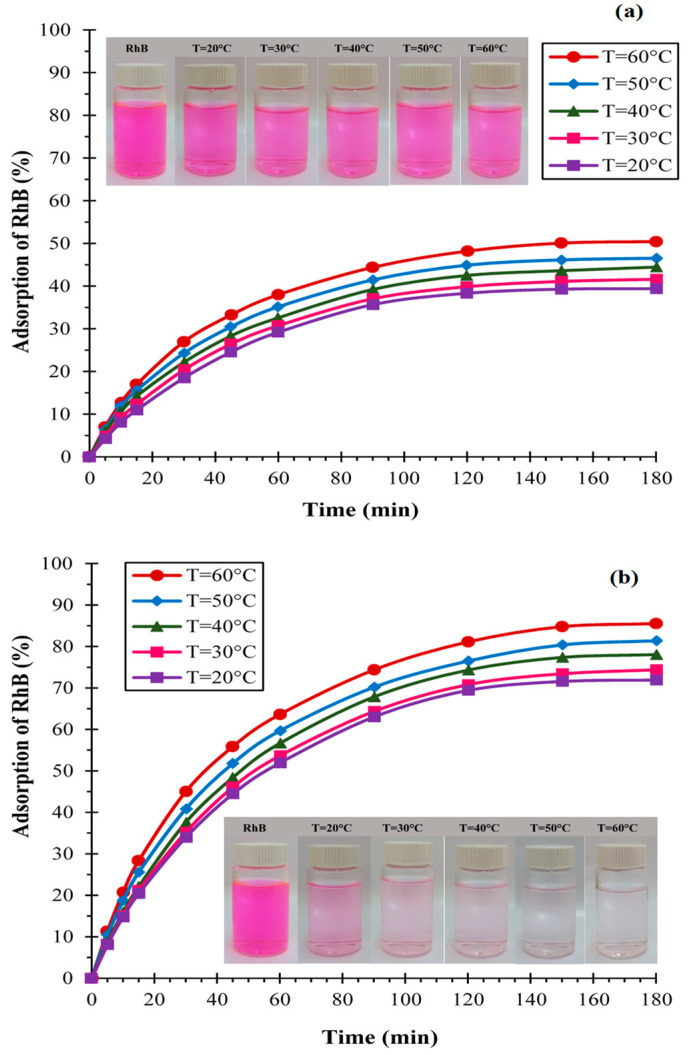
Evolution of the adsorption of RhB dye at different temperatures on (**a**) KS44-0 and (**b**) KS44-20.

The experiments were performed with the carbon adsorbents KS44-20 and KS44-0 using a 10 mg/L initial dye concentration at different temperatures of 20, 30, 40, 50 and 60 °C (Figure 3). The percentage removal of RhB increased with the increase in temperature up to 60 °C. In fact, the adsorption increased from 71 to 85% and from 39 to 51% for KS44-20 and KS44-0, respectively. The kinetic energy of the adsorbent molecules was therefore affected by the RhB solution’s temperature, which could result in a greater adsorption capacity on the surfaces and in the pores of both carbon composites (KS44-0 and KS44-20). Due to intermolecular forces of attraction between a solid and the material adsorbed, porous adsorbents favor the probability of adsorbate diffusion [15]. As a result, the adsorption process seems to be controlled by adsorbate diffusion and mass transfer onto different porous adsorbents, which is also favored as temperature rises. The adsorption process is likewise endothermic for RhB dye, as shown by the preceding data [16].

#### 3.1.3. Adsorption Kinetics

**Figure 4:**
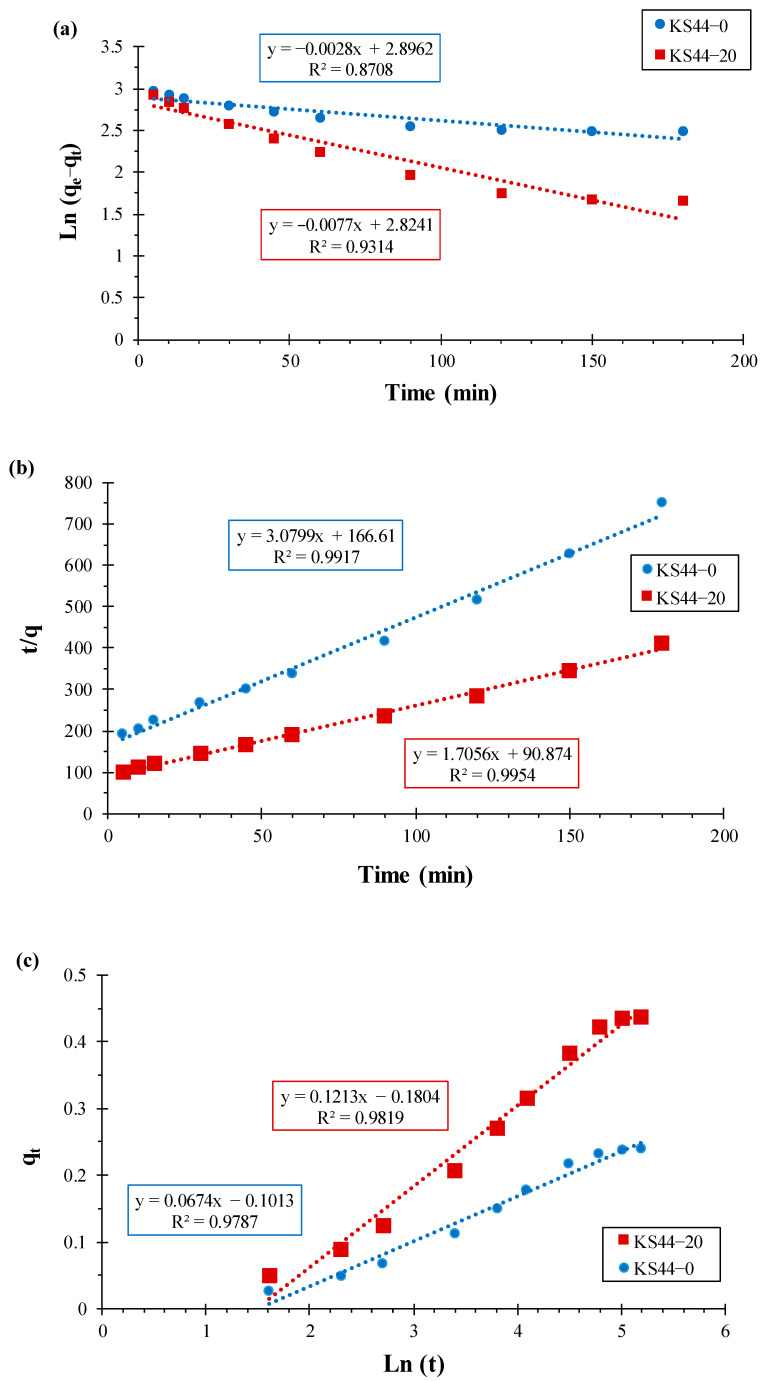
Kinetic parameters of the pseudo-first-order (**a**), pseudo-second-order (**b**) and the Elovich model (**c**) for RhB adsorption on KS44-0 and KS44-20.

The experimental adsorption data were analyzed using the pseudo-first-order [17,18,19] and pseudo-second-order [17,18,19] Elovich models [20,21] to explore the kinetics of the RhB adsorption process over carbon composites (KS44-0 and KS44-20). Different linear form calculations were used (Equations (3)–(6)) and are presented in Figure 4.

The pseudo-first-order kinetic rate expression of Lagergren is in the form of:

(3) $$\frac{1}{q_{t}}=\frac{k_{1}}{q_{1\;}\times t}+\frac{1}{q_{1}}$$

where *q_t_* and *q_e_* (mg.g^−1^) are the adsorbed amounts of RhB at time t and at equilibrium, respectively, and *k*_1_ (min^−1^) is the adsorption rate constant. The pseudo-first-order model was examined by the extrapolation of the plot ln (*q_e_*–*q_t_*) vs. (time), according to Equation (4):

(4) $$\ln\;\left(q_{e}-q_{t}\right)=\;\ln\;\left(q_{e}\right)-\frac{k_{1}}{2.303}\times t$$

The pseudo-second-order kinetics was examined by the extrapolation of the plot (*t*/*q_t_*) vs. (time), according to Equation (5):

(5) $$\frac{t}{q_{t}}=\frac{1}{\left(k_{2}\times q_{e}{}^{2}\right)}+\frac{t}{q_{e}}$$

where *q_e_* (mg.g^−1^) is the maximum adsorption capacity and *k*_2_ (g.mg^−1^.min^−1^) is the rate constant of adsorption.

The Elovich model can be described in a linear form as defined by Equation (6):

(6) $$q_{t}=\frac{1}{\beta }\;Ln\left(\alpha \beta \right)+\frac{1}{\beta }\;Ln\left(t\right)$$

where *q_t_* is the amount adsorbed at time t (mg.g^−1^), α represents the initial adsorption rate (mg.g^−1^.min^−1^) and *β* is related to the desorption constant for each experiment (g.mg^−1^).

The extracted adsorption parameters of the various kinetic models from Figure 4 are tabulated in Table 2, from which it is clear that the correlation coefficient (R^2^), indicating the proximity of the results, shows the mechanism under study. When the value is close to 1, it is evident that the reaction is more likely to follow it. The pseudo-second-order model revealed a better fit to the adsorption data, in which the calculated values of qe (0.249 and 0.452 mg.g^−1^) were the closest to the experimental values of *q_e_* _(exp)_ (0.239 and 0.437 mg.g^−1^) for the KS44-0 and KS44-20 composites, respectively. This demonstrated that the adsorption kinetics of RhB dye onto carbon composites is better described by a pseudo-second-order model.

#### 3.1.4. Adsorption Mechanism

The intra-particle diffusion [7,20] and Bangham models were applied to identify the adsorption mechanism. According to this concept, the intra-particle diffusion model developed by Weber and Morris has been widely applied to examine the rate-limiting step during adsorption [22], and is given by the following equation:

(7) $$q_{t}=k_{id}\times t^{\frac{1}{2}}+A$$

where *q_t_* (mg.g^−1^) is the amount of RhB adsorbed at time *t*, *k_id_* (mg.g^−1^ min^−1/2^) is the rate constant for intra-particle diffusion and *A* is a constant.

Kinetic data were further used to determine the controlling step occurring in the adsorption system using Bangham model that is based on the assumption that pore diffusion takes place during adsorption [23,24], in the following form:

(8) $$\log\;(\frac{C_{0}}{C_{0\;}-q_{t\;}\times m})=\log\;(\frac{K_{B}}{2.303\times V})+\alpha \;Ln\;\left(t\right)$$

where *C*_0_ (mg.L^−1^) is the initial concentration of dye solution, *V* (mL) is the volume of solution, *q_t_* (mg.g^−1^) is the amount of dye adsorbed at time t, m (g.L^−1^) is the weight of adsorbent per liter of solution and (α < 1) and *K_B_* are constants.

**Figure 5:**
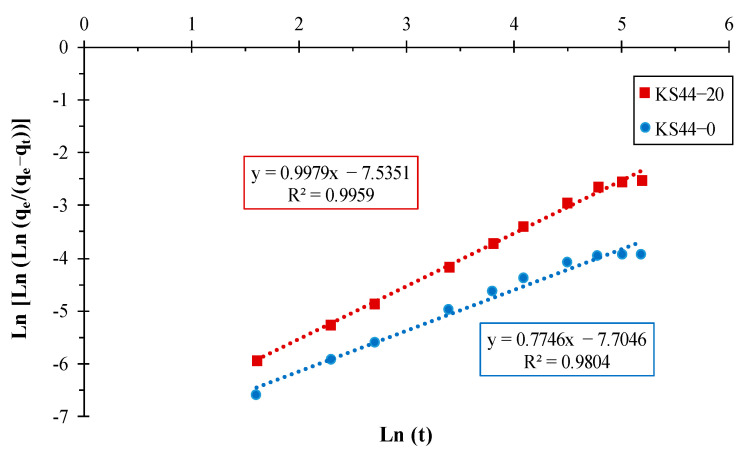
Intra-diffusion kinetic model representation for RhB adsorption on (**a**) KS44-0 and (**b**) KS44-20.

**Figure 6:**
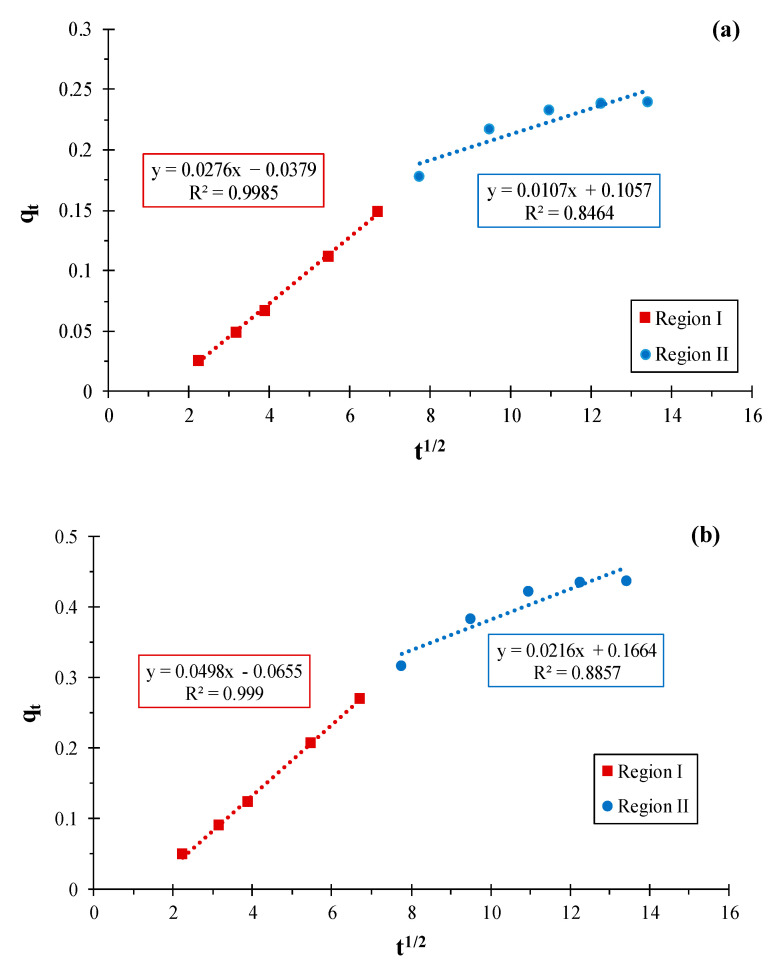
Bangham kinetic model representation for RhB adsorption on (**a**) KS44-0 and (**b**) KS44-20.

The kinetic parameters for the adsorption of RhB onto carbon composites were determined using the slope-intercept form (Figure 5) and are summarized in Table 3. The graphical representation of q_t_ versus t^1/2^ that describes model dependencies obtained for RhB dye adsorption (Figure 5) did not pass through the origin. Next, there were two separate linear regions independently evaluated in both cases. From this figure, it is evident that intra-particle diffusion is a significant step in the process of the adsorption of RhB onto KS44-0 and KS44-20, especially in the first 60 min of the experiment. Figure 5 shows a linear plot of kinetic data, not passing through the origin, which indicates that the diffusion of adsorbate into the pores of adsorbents is not the only rate-controlling step. Nonetheless, the Bangham plot’s linearity indicates that more than one process may be at work when it comes to the uptake of RhB onto carbon composite materials (Figure 6). As indicated in Table 3, the correlation coefficients (R^2^) of the intra-particle diffusion and the Bangham model converge to 1, which indicates the applicability of these two adsorption kinetic models. Thus, the intra-particle diffusion model, which is highly supported by the Bhangam model, is the second-best model to produce a decent match to the experiment, followed by a pseudo-second-order. This suggests that the data fit and mainly control the adsorption process of RhB onto carbon composites with three kinetic models for all experimental conditions.

### 3.2. Adsorption Isotherm Study

**Figure 7:**
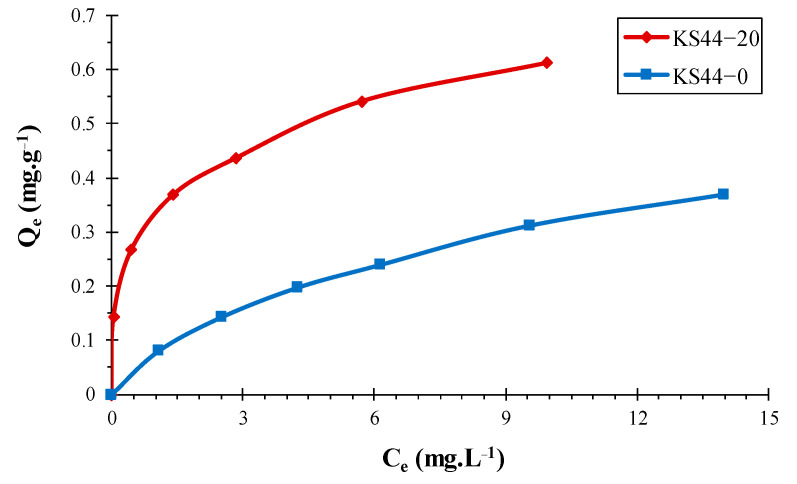
Adsorption isotherms of RhB on carbon composites (**a**) KS44-0 and (**b**) KS44-20. (T = 20 °C, pH = 3, and time = 3 h).

The adsorption isotherms of RhB dye on KS44-0 and KS44-20 composites at room temperature were studied (Figure 7). Indeed, the adsorption isotherm describes the interaction of dyes with adsorbents, which are determinant factors for the performance of adsorption processes. According to the concentration of adsorbate remaining in solution C_e_ (in mg.L^−1^), it expresses the amount of adsorbate on the adsorbent qe (in mg.g^−1^ of adsorbent). The adsorption isotherms indicate that the amount of adsorbed RhB increased with the increase in initial concentration C_e_. The results demonstrate that the adsorption occurred quickly in the early stages of the experiment before processing slowed down to reach a plateau, a sign that porous composite surfaces are saturated. In the low Ce range, the adsorption of RhB adsorbent on KS44-20 was closer to the theoretical line, corresponding to 100% adsorption, which suggests a higher binding affinity of RhB dye onto KS44-20.

**Figure 8:**
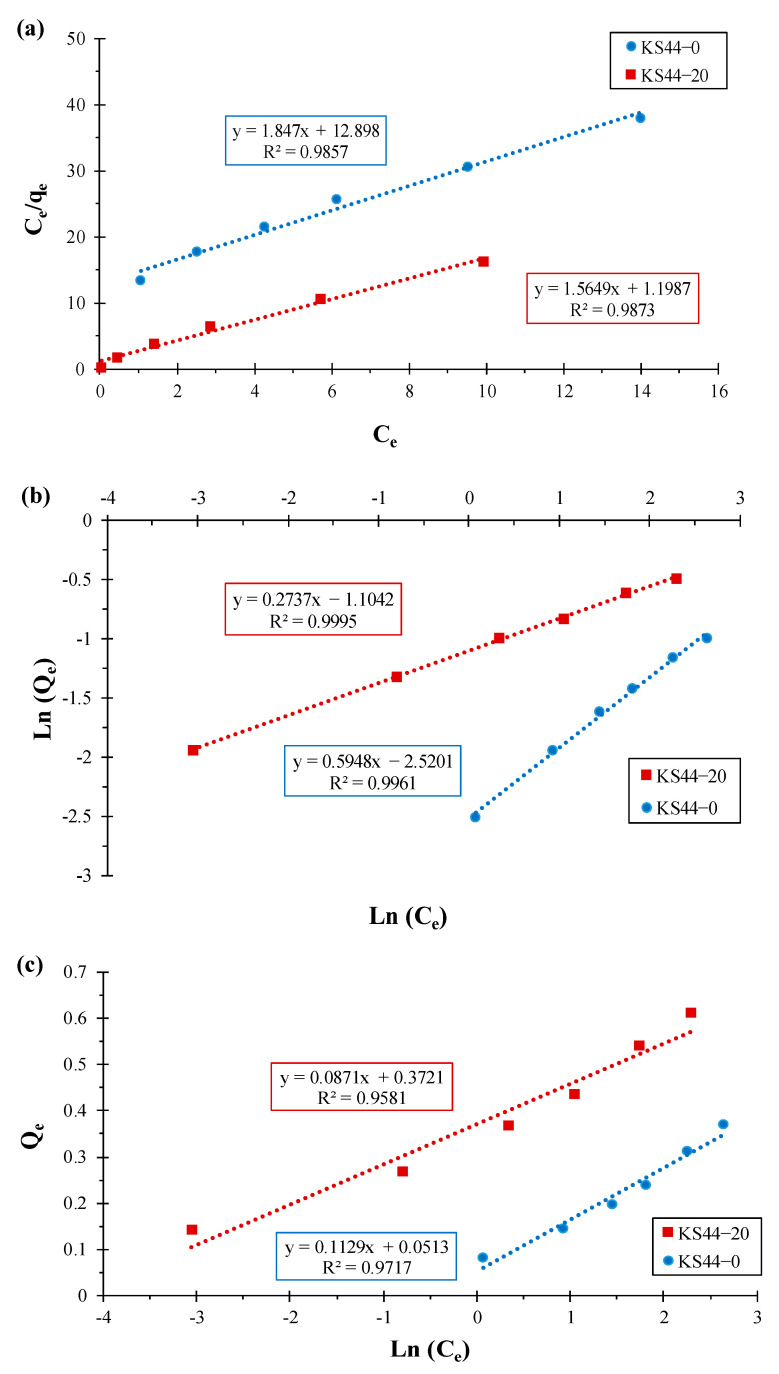
Transformed adsorption isotherm for (**a**) the Langmuir model, (**b**) the Freundlich model and (**c**) the Temkin model for RhB adsorption on KS44-0 and KS44-20 composites.

The equilibrium nature of adsorption has been described by a number of isotherm equations. Adsorption data were fitted to the Langmuir [6,9], Freundlich [20,21] and Temkin [21,25] equations, and the isotherm parameters were calculated and reported accordingly (Figure 8). The linear transformation of the Langmuir adsorption isotherm, assuming that the adsorbate adsorption is limited to one molecular layer and the surface of the adsorbent is homogeneous, is given in Equation (9):

(9) $$\frac{C_{e}}{q_{e}}=\frac{C_{e}}{q_{max}}+\frac{1}{\left(q_{max}\times K_{L}\right)}$$

where *K_L_* (L.mg^−1^) is the Langmuir adsorption constant related to the energy of adsorption, *q_e_* (mg.g^−1^) is the equilibrium adsorbate amount on the adsorbent, *C_e_* (mg.L^−1^) is the equilibrium dye concentration and *q_max_* (mg.g^−1^) is the maximum adsorption retained by the adsorbent at monolayer coverage [26,27,28].

The linear transformation of the Freundlich adsorption isotherm, which reflects the description of multilayer adsorption with interactions between adsorbed molecules and heterogeneous surfaces, is given as Equation (10):

(10) $$ln\;(q_{e})=\left(\frac{1}{n}\right)\times ln\;(C_{e})+ln\;(K_{F})$$

where *K_F_* is the Freundlich adsorption capacity and (1/*n*) is the Freundlich adsorption intensity.

The Temkin adsorption isotherm’s linear transformation, which does not only examine the effects of some indirect adsorbate/adsorbent interactions on the adsorption process but also assumes that the heat of adsorption of all molecules would decrease linearly with the coverage of the adsorbed molecule layer, is given as Equation (11):

(11) $$q_{e}=B_{T}\;ln\;(K_{T})+B_{T\;}ln\;(C_{e})$$

where *B_T_* (J.mol^−1^) is the Temkin adsorption constant and *K_T_* (L.mg^−1^) is the Temkin isotherm constant.

Table 4 provides an overview of the parameters and correlation coefficients of the Langmuir, Freundlich, and Temkin isotherm models used to study RhB adsorption on KS44-0 and KS44-20. The Freundlich model yielded the highest correlation coefficients of the data fitting (R^2^ > 0.99). Therefore, the Freundlich model, which describes multilayer adsorption (more than one layer of molecules), in which adsorbed molecules are bound by hydrophobic or other weak interactions, provides a good representation of the adsorption isotherm of RhB dye from carbon composites [29,30,31].

### 3.3. Adsorption Thermodynamics

Thermodynamic parameters such as standard Gibbs free energy change (ΔG°_ads_), standard enthalpy change (ΔH°_ads_) and standard entropy change (ΔS°_ads_) were determined using the following equations [32,33,34]:

(12) $$K_{D}=\frac{q_{e}}{C_{e}}$$

(13) $$\Delta G{}^{\circ}=-\;R\;T\times \mathrm{Ln}\;\left(K_{d}\right)$$

(14) $$\mathrm{Ln}\;\left(K_{d}\right)=\frac{\Delta S{}^{\circ}}{R}-\frac{\Delta H{}^{\circ}}{R\;T}$$

(15) $$\Delta G{{}^{\circ}}_{\mathrm{ads}}=\Delta H{{}^{\circ}}_{\mathrm{ads}}-T\;\times \;\Delta S{{}^{\circ}}_{\mathrm{ads}}$$

where R (8.314 J.K^−1^.mol^−1^) is the universal gas constant, T (K) is the absolute temperature scale, K_D_ (L.g^−1^) is the equilibrium dissociation constant and C_e_ (mg.L^−1^) and qe (mg.g^−1^) are the equilibrium concentration of dye in solution and the amount of dye adsorbed onto the adsorbent at equilibrium, respectively.

**Figure 9:**
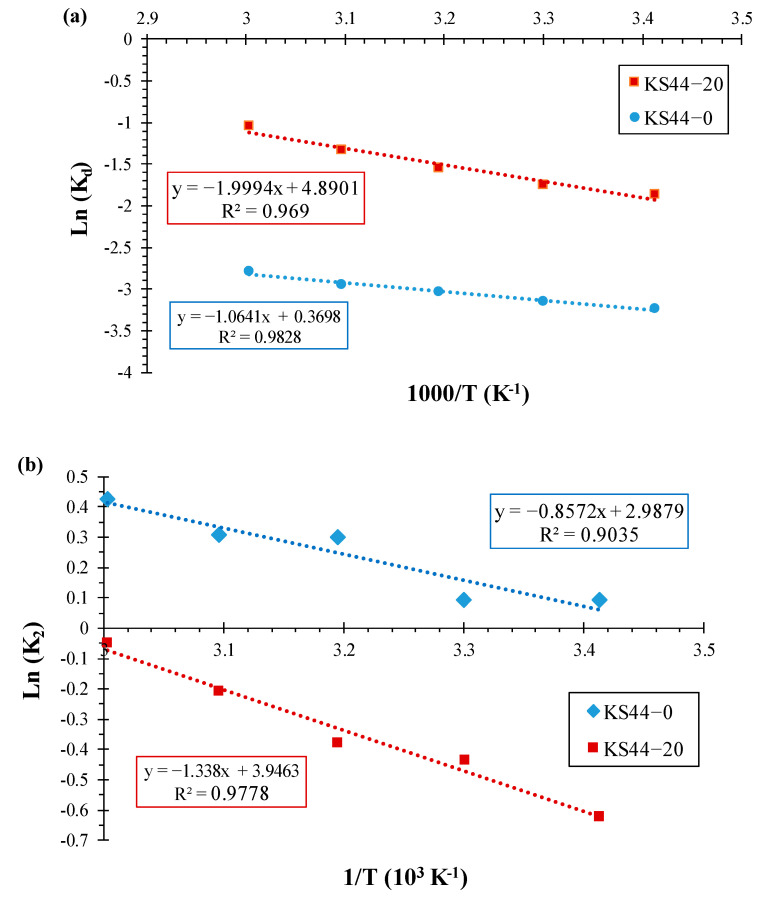
(**a**) The Van’t Hoff plots and (**b**) the Arrhenius plot for the determination of activation energy for adsorption of RhB on KS44-0 and KS44-20 at different temperatures.

The values of ∆H°_ads_ and ∆S°_ads_ were obtained from the linear Van’t Hoff plots of ln (K_D_) versus 1/T [35,36,37] (depicted in Figure 9a), and the thermodynamic parameters obtained for the adsorption process are summarized in Table 5.

The influence of temperature on the retention rate of the RhB dye on the adsorbents (KS44-0 and KS44-20) is illustrated in Figure 9a. According to the adsorption theory, the adsorption rate and adsorption capacity increase with the increase in temperature (from 20 °C to 60 °C). In accordance with the first law of thermodynamics, this phenomenon does not only suggest that the reaction of RhB dye adsorption on composites is endothermic (ΔH°_ads_ > 0), but also explains why reactions occur more rapidly at higher temperatures [38,39].

From Table 5, the positive values of standard enthalpy change (∆H°_ads_) reveal the endothermic nature of the adsorption process that absorbs thermal energy from its surroundings, namely heat transfer into the system.

This is in accordance with the increase in adsorption capacity upon increasing temperature. The heat evolved during physical adsorption is of the same order of magnitude as that of condensation, (i.e., 2.1–20.9 kJ·mol^−1^), while the heat of chemisorption generally falls into the range of 80–200 kJ·mol^-1^ [40,41,42,43]. Hence, the values of standard enthalpy change (ΔH°_ads_ = 8.846 and 16.623 kJ·mol^−1^) were less than 20.9 kJ·mol^−1^ for KS44-0 and KS44-20, respectively. This indicates that RhB dye adsorption by using carbon composites is attributed to a physical adsorption process rather than a chemical adsorption process. As a result of this process, the second law of thermodynamics states that the positive values of standard entropy change (∆S°_ads_) reflect an increase in disorder at the adsorbate/adsorbent interface in the adsorption process and reveal the binding affinity of the adsorbate to adsorbent materials toward RhB dye molecules. For a full understanding of this alloying effect, the increase in the standard entropy change (∆S°_ads_) can provide a larger number of adsorption sites. It can also process more fixation binding sites, allowing the adsorption of RhB dye, which increases the RhB adsorption capacity as the surface and internal structure of the porous materials become more fully saturated by the adsorbate. The thermodynamic evidence of the viability and spontaneity of the RhB dye adsorption process on KS44-0 and KS44-20 composite materials is provided by the negative values of standard Gibbs free energy change (∆G°_ads_) at different temperatures (from 20 °C to 60 °C).

**Figure 10:**
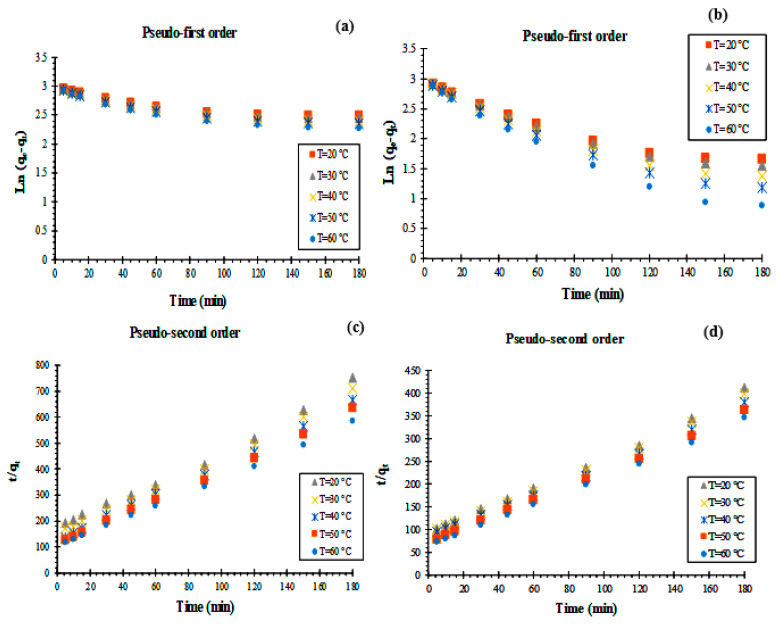
Pseudo-first-order and pseudo-second-order models plot for RhB at different temperatures onto (**a**,**c**) KS44-0 and (**b**,**d**) KS44-20.

The expression of Arrhenius’ law reveals the activation energy (E_a_) for the dye adsorption by using the pseudo-second-order rate constant (Figure 10), as given by Equation (16) [44,45]:

(16) $$Ln\;(K_{2})=Ln\;\left(A_{0}\right)-\frac{E_{a}}{R\;T}$$

where *K*_2_ is the rate constant for the reaction at a given temperature (L.g^−1^), A_0_ is the temperature-independent factor (g.mg^−1^.min^−1^), *E_a_* is the activation energy (kJ·mol^−1^), R is the universal gas constant (8.314 kJ·mol^−1^) and T is the solution temperature (K).

The activation energy was determined from the slope of the straight line of plotting ln K_2_ versus 1/T (Figure 9b). If the value of activation energy requirement is small (E_a_ = 0–40 kJ·mol^−1^), and since the forces involved are weak, the adsorption process flows by chemical ion exchange. If the activation energy *E_a_* < 40 kJ·mol^−1^, then the adsorption process is physical in nature, whereas if the *E_a_* value is within the range of 40–800 kJ·mol^−1^, then the adsorption process is chemisorption in nature and involves forces much stronger (chemical bonds) than physical adsorption [46,47,48,49,50]. Therefore, the values of activation energy (*E_a_*) (i.e., minimum energy required for a reaction to occur), calculated using the Arrhenius equation (E_a_ = 7.126 and 11.124 kJ·mol^−1^), are less than 40 kJ·mol^−1^ for KS44-0 and KS44-20, respectively (Table 6). This implies that the adsorption process is controlled by a physisorption interaction mechanism, and thus, activation energy requirements are small (below 40 kJ·mol^−1^). These data were consistent with other results from several investigations, which suggested that the adsorption has a multilayer character and involves weak intermolecular forces [51,52,53,54].

**Figure 11:**
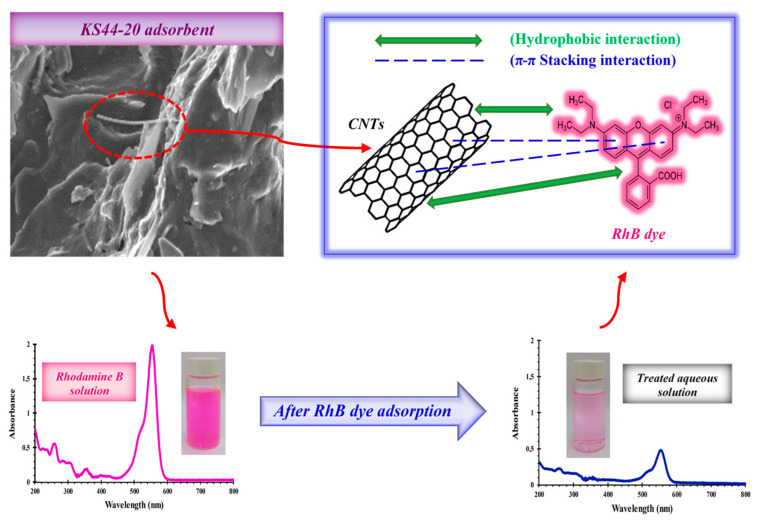
Mechanism of adsorption for the removal of RhB dye onto carbon composites ((RhB) = 10 mg.L^−1^, T = 20 °C, pH = 3 and time = 3 h.

It is worthwhile mentioning that RhB dye solution has two primary distinctive absorption bands (Figure 11). The ultraviolet (UV) light spans a range of wavelengths between 200 and 400 nm, which represents the aromatic rings of RhB (π → π* transition of C=C, C=O and C=N groups). The visible radiation bands at 555 nm (n → π* transition of C=N and C=O groups) are attributable to the color of dye solution, which was used for the monitoring of dye decolorization [55]. The high adsorption performance of the carbon graphite/carbon nanotube composite material towards RhB dye might be explained by the feasible adsorption mechanism involved in the adsorption process (Figure 11). This startling discovery is especially pertinent to the π–π stacking interactions, which corresponds to a very small binding energy between the aromatic backbone of the RhB dye molecules and the large delocalized π-electrons over the entire hexagonal skeleton of carbon graphite and carbon nanotubes [56,57,58,59]. Moreover, the observed porosities emanate from the pores available for RhB dye uptake, which further enhances the absorption capacity in the carbon composites. These pores provide excellent surfaces for trapping and thus favor the RhB dye molecules’ adsorption [60,61,62].

### 3.4. Comparison of Performances with Other Adsorbents

The results of the adsorption capacities and thermodynamic parameters for the adsorption of RhB dye onto carbon composites were compared with other adsorbents with the same dye reported in the literature, as displayed in Table 7. Many of the reported research works have investigated the removal of RhB using carbon-based materials as adsorbents, such as terpyridine functionalized magnetic nanoparticles (MNP-Tppy), clay–cellulose composites, fly ash, commercial activated carbon (CAC), activated carbon obtained from pericarp of rubber fruit (PrAC), graphene oxide (GO) and multi-walled carbon nanotubes (MWCNTs). However, it is obvious that the adsorption capacities of the as-prepared carbon graphite/carbon nanotubes composite regarding RhB have higher RhB removal abilities than the previously mentioned adsorbents. The above study reveals that KS44-20 is a promising adsorbent for the removal of RhB from aqueous solutions due to the presence of carbon nanotubes, high surface area and high adsorption capacity. Therefore, the carbon graphite/carbon nanotube composite material has great potential for wastewater treatment and has been proven to be better than other adsorbents reported in the literature.

## 4. Conclusions

Porous carbon composite materials (KS44-0 and KS44-20) have been used for the absorption of Rhodamine B (RhB) dye from aqueous solution. The removal effectiveness of carbon nanotubes as adsorbents for RhB from aqueous solution, as well as cationic dyes, was described in this study, which aimed to limit the environmental effect of synthetic product dyes in water and wastewater. Eventually, the results showed that the surface characteristics of carbon materials were substantially altered by the addition of ferrocene content, related to the presence of carbon nanotubes (CNTs) within the composite material (KS44-20). The presence of CNTs that grew on the surface and inside the pores gave rise to more active sites and surface area for the interaction between the cationic dye, Rhodamine B (RhB). Hence, this higher adsorption for the adsorbent (KS44-20) relative to (KS44-0) can be explained by the fact that carbon nanotubes have been investigated as interesting and high-performing adsorbent materials to be used in water treatment. The optimization of the adsorption process depends on large parameters (time, pH, temperature, the concentration of the pollutant and the nature of the adsorbent), which govern its effectiveness. In such a way, by keeping the pH constant at 3.0 and varying the solution temperatures at 20 °C, 30 °C, 40 °C, 50 °C and 60 °C, one could find a significant influence on the adsorption process for RhB dye. The best Rhodamine B dye removal effectiveness was found in almond shells (85%), which took place within 3 h for graphite carbon/carbon nanotube composites. The equilibrium adsorption data fitted well to the Freundlich adsorption isotherm, better than the Langmuir isotherm. The pseudo-second-order kinetics model was observed to fit the adsorption data. At all temperatures, negative standard Gibbs free energy change values (ΔG°_ads_) indicated that the adsorption process was endothermic, spontaneous and practicable. The values of standard enthalpy change (ΔH°_ads_) and activation energy (E_a_) indicated that the adsorption process is physical sorption. As a result, RhB dye is a planar molecule that is readily adsorbed due to the π–π interactions between the dyes’ aromatic backbones and the hexagonal skeleton of graphite carbon and carbon nanotubes. Thus, it is believed that carbon composites will play a major role in organic pollutant reduction. The prepared adsorbent efficiency was compared to other adsorbents regarding the same dye removal, and we may conclude that its action is enhanced compared to other adsorbents reported the in the literature.

## Figures and Tables

**Table 1:** Textural parameters of KS44-0 and KS44-20 detected with BET method and mercury intrusion porosimetry technique [12].

Composites	Porosity (%)	Average Pore Diameter (4 V/A) (nm)	BET Surface Area (m^2^/g)
**KS44-0**	45.65	102.20	85.67
**KS44-20**	52.08	150.20	131.47

**Table 2:** Kinetic parameters for RhB dye adsorption on KS44-0 and KS44-20: (C_0_ = 10 mg.L^−1^, pH = 3 and T = 20 °C).

Composite	Pseudo-First-Order			Pseudo-Second-Order			Elovich			q_e (exp)_ (mg.g^−1^)
	k_1_ (min^−1^)	q_e (cal)_ (mg.g^−1^)	R^2^	k_2_ (g.mg^−1^.min^−1^)	q_e (cal)_ (mg.g^−1^)	R^2^	β (g.mg^−1^)	α mg.g^−1^.min^−1^)	R^2^	
KS44-0	0.002	18.105	0.870	1.095	0.249	0.991	0.067	0.101	0.978	0.239
KS44-20	0.007	16.845	0.931	0.536	0.452	0.995	0.121	0.180	0.981	0.437

**Table 3:** Kinetic parameters obtained from intra-particle diffusion and Bangham models for RhB adsorption on KS44-0 and KS44-20.

Composites	Intra-Particle Diffusion Model						Bangham Model		
	Region I			Region II					
	k_dif_ (min^−1^)	C (mg.L^−1^)	R^2^	k_dif_ (min^−1^)	C (mg.L^−1^)	R^2^	K_B_ (min^−α^)	A	R^2^
**KS44-0**	0.027	0.037	0.998	0.010	0.105	0.846	0.774	−7.704	0.980
**KS44-20**	0.049	0.065	0.999	0.021	0.166	0.885	0.997	−7.535	0.995

**Table 4:** Langmuir, Freundlich and Temkin isotherm model parameters for RhB adsorption on KS44-0 and KS44-20 composites.

Composites	Langmuir Isotherm			Freundlich Isotherm			Temkin Isotherm		
	q_max_ (mg.g^−1^)	K_L_ (L.mg^−1^)	R^2^	K_F_ (mg.g^−1^)	n	R^2^	B_T_ (J.mol^−1^)	K_T_ (L.mg^−1^)	R^2^
**KS44-0**	0.541	6.983	0.985	0.080	1.681	0.996	0.112	1.575	0.971
**KS44-20**	0.639	0.765	0.987	0.331	3.653	0.999	0.087	71.671	0.958

**Table 5:** Thermodynamics parameters for the adsorption of RhB onto KS44-0 and KS44-20.

Composites	ΔH° (kJ·mol^–1^)	ΔS° (J.mol^–1^)	ΔG° (kJ·mol^–1^)					R^2^
			T = 20 °C	T = 30 °C	T = 40 °C	T = 50 °C	T = 60 °C	
**KS44-0**	9.079	4.010	−1.165	−1.206	−1.246	−1.286	−1.326	0.982
**KS44-20**	16.747	41.583	−1.158	−1.198	−1.238	−1.278	−1.318	0.969

**Table 6:** Determined activation energies of the adsorption of RhB onto KS44-0 and KS44-20.

Composites	Activation Energy (E_a_) (kJ·mol^–1^)	R^2^
**KS44-0**	7.126	0.903
**KS44-20**	11.124	0.977

**Table 7:** Comparison of the Rhodamine B (RhB) dye adsorption capacities and thermodynamic parameters on different carbon adsorbents.

Carbon-Based Materials	Conditions				Qe (mg.g^−1^)	Kinetic Model	Isotherm Model	ΔH°_ads_ (kJ·mol^−1^)	ΔG°_ads_ (kJ·mol^−1^)	ΔS°_ads_ (kJ·mol^−1^)	E_a_ (kJ·mol^−1^)	Ref
	(RhB) (mg.L^−1^)	pH	T (°C)	Time (h)								
**MNP-Tppy**	10	6	20	4	4.742	Pseudo-second-order	Langmuir	0.937	−1.220	3.69	-	[63]
**Cellulose–clay**	10	2	30	2	2.789	Pseudo-second-order	Redlich–Peterson	0.911	−3055.14	10.086	-	[64]
**Fly ash**	10	7	30	3	0.334	Pseudo-first-order	Langmuir and Freundlich	−24.97	−9.49	51.05	-	[65]
**CAC**	150	4	30	12	0.428	Pseudo-second-order	Langmuir	4.01	-	-	-	[8]
**PrAC**	300	4	30	36	0.110	Pseudo-second-order	Langmuir	14.81	-	-	-	[8]
**GO**	67	7	25	24	0.043	-	Freundlich	4.18	−1.77	5.95	-	[66]
**MWCNTs**	10	1	25	0.5	0.148	Pseudo-second-order	Langmuir and Freundlich	-	-	-	-	[67]
	10	7	25	0.5	0.109							
	10	10	25	0.5	0.127							
**KS44-0**	10	3	20	3	0.239	Pseudo-second-order	Freundlich	9.079	−1.165	4.010	7.126	**This work**
**KS44-20**	10	3	20	3	0.437	Pseudo-second-order	Freundlich	16.747	−1.158	41.583	11.124	

## Data Availability

The data presented in this study are available within the paper itself and the supplementary material. Any more data needed is available on request from the corresponding author because it is kept in the laboratory note books.

## Supplementary Materials

The following supporting information can be downloaded at: https://www.mdpi.com/article/10.3390/ma16031015/s1, Figure S1: Calibration curve (at 555 nm) and chemical structure of Rhodamine B dye molecule (RhB).
