# Supplementary material for: Adsorptive Removal of Rhodamine B Dye Using Carbon Graphite/CNT Composites as Adsorbents: Kinetics, Isotherms and Thermodynamic Study
Source: Materials (Basel). 2023 Jan 22;16(3):1015. doi: 10.3390/ma16031015 (PMC9920111; doi:10.3390/ma16031015)
Supplement: Supplementary file 1 [file materials-16-01015-s001.zip › materials-2130358-supplementary.pdf]

materials

MDPI

Supplementary Materials

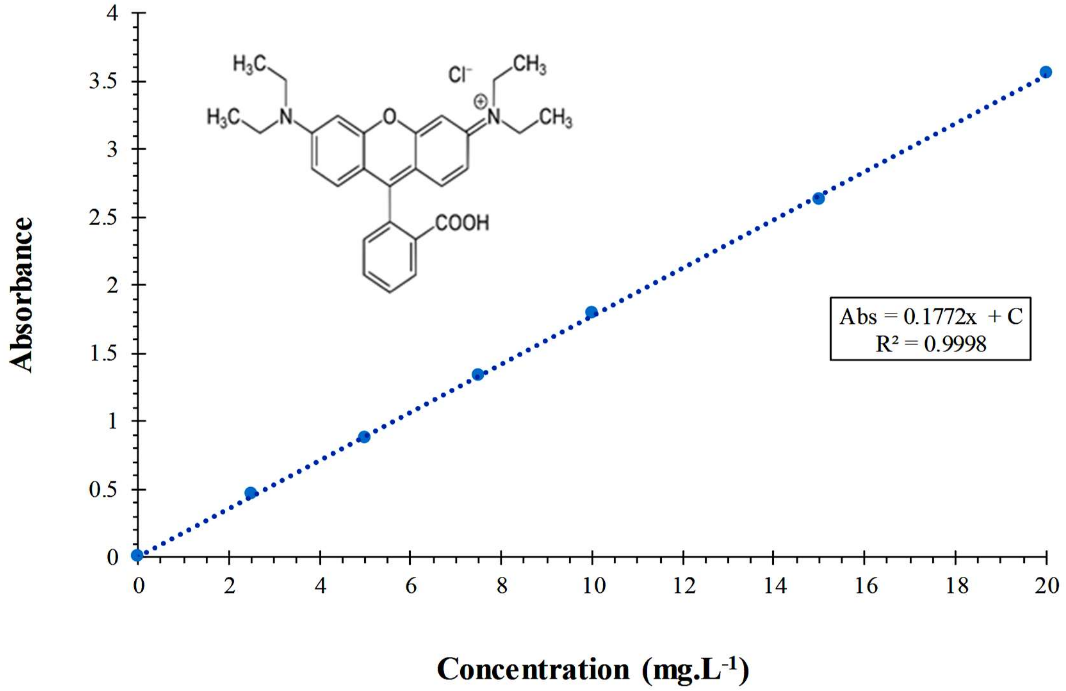

Figure S1. Calibration curve (at 555 nm) and chemical structure of Rhodamine B dye molecule (RhB).
